# Design and Reality-Based Modeling Optimization of a Flexible Passive Joint Paddle for Swimming Robots

**DOI:** 10.3390/biomimetics9010056

**Published:** 2024-01-19

**Authors:** Junzhe Hu, Yaohui Xu, Pengyu Chen, Fengran Xie, Hanlin Li, Kai He

**Affiliations:** 1 Shenzhen Institute of Advanced Technology, Chinese Academy of Sciences, Shenzhen 518055, China; hujz@mail.uc.edu (J.H.); yh.xu@siat.ac.cn (Y.X.); hl.li1@siat.ac.cn (H.L.); 2 Chongqing University-University of Cincinnati Joint Co-op Institute, Chongqing University, Chongqing 400044, China; pychen@cqu.edu.cn; 3 School of Artificial Intelligence, Shenzhen Polytechnic, Shenzhen 518055, China; xiefengran@szpt.edu.cn

**Keywords:** flexible passive joint paddle, modeling optimization, soft robot, bionic robot

## Abstract

Rowing motion with paired propellers is an essential actuation mechanism for swimming robots. Previous work in this field has typically employed flexible propellers to generate a net thrust or torque by using changes in the compliance values of flexible structures under the influence of a fluid. The low stiffness values of the flexible structures restrict the upper limit of the oscillation frequency and amplitude, resulting in slow swimming speeds. Furthermore, complex coupling between the fluid and the propeller reduce the accuracy of flexible propeller simulations. A design of a flexible passive joint paddle was proposed in this study, and a dynamics model and simulation of the paddle were experimentally verified. In order to optimize the straight swimming speed, a data-driven model was proposed to improve the simulation accuracy. The effects of the joint number and controller parameters on the robot’s straight swimming speed were comprehensively investigated. The multi-joint paddle exhibited significantly improved thrust over the single-joint paddle in a symmetric driving mode. The data-driven model reduced the total error of the simulated data of the propulsive force in the range of control parameters to 0.51%. Swimming speed increased by 3.3 times compared to baseline. These findings demonstrate the utility of the proposed dynamics and data-driven models in the multi-objective design of swimming robots.

## 1. Introduction

Aquatic animals are adept at using passive changes in their body structures to adapt to or enhance their interactions with fluids, generating complex behaviors from simple movement patterns [1,2]. Flexible propellers can guide the fluid towards the preferred axis without much loss [3], with simple control modes and high mechanical efficiency [4,5]. This has motivated researchers to take a great interest in flexible propellers. The use of pairs of flexible propellers undergoing paddling motion to generate positive net thrust is a common and efficient swimming mode. The motion cycle, which consists of a recovery stroke (RS) to reset the propellers and a power stroke (PS) to generate positive net thrust, has been extensively studied in the last decade. Previous studies have shown that flexible propellers using drag forces can construct a positive net thrust by using asymmetric forces in the RS and PS to achieve positive displacement [6,7,8]. Some researchers have also designed asymmetric flexible propellers using bendable structures or deformable appendages to influence their thrust through the difference in the force projection areas of the propellers in the RS and PS [9,10,11,12]. However, current propellers in this field usually suffer from small deformation amplitudes of the structures of the flexible propellers, resulting in small morphological differences between the PS and RS [13,14,15]. The resulting low net thrust generated leads to slow movement of the swimming robot, which relies on asymmetric control modes in the PS and RS to achieve effective movement, adding to the control complexity.

**Figure 1:**
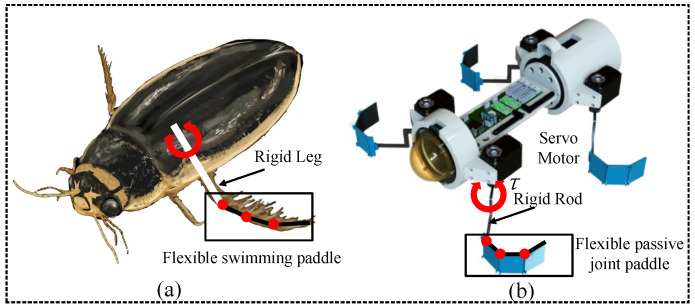
The bionic design of swimming robot. (**a**) The diving beetle and the structure of its flexible swimming legs. (**b**) The swimming robot and flexible passive joint paddle inspired by diving beetles.

We found that diving beetles possess the ability to move at high speeds underwater due to their highly articulated swimming feet, which use changes in the contours of their limb movements to produce an asymmetric gait for propulsion throughout the movement cycle [16,17], allowing for a high propulsion efficiency of 84%. Inspired by this use of passive changes in the motion profile to generate thrust in the propulsion cycle, a series of flexible passive joint paddles (FPJPs) for flexible propellers were designed in this study, which allowed asymmetric changes of the paddle morphology in the RS and PS through nonlinear compliance constructions at the joints to generate a net thrust. The design of FPJP adopted a passive concept to achieve better coordination. This approach is preferred because it provides simplicity in fabrication and control as well as higher propulsion efficiency compared to existing work. Swimming robots equipped with FPJPs as propellers can use uniform symmetric driving modes to achieve complex motions and effective fast movements [18,19]. The flexible passive joint paddle and swimming robot inspired by diving beetle are shown in Figure 1.

To fully utilize the propulsive forces generated by the passive structure in the fluid and improve the controllability of the deformation caused by the passivity of the flexible propellers, a highly accurate dynamics model is needed to understand and analyze the structural changes, dynamic motions, fluid–solid interactions, and propulsive coupling of the asymmetric structure. In this study, we used a pseudo-rigid body model (PRBM) [20,21] to model the FPJP as a series of pseudo-rigid body links connected by torsion springs, combined with blade element theory to calculate the hydrodynamic forces. We ran simulations using Simulink to obtain data about the motion state of each point on the paddle and the hydrodynamic forces, which were modeled in a manner consistent with the joint characteristics of the highly articulated legs of diving beetles [22]. Due to the overly complex coupling between the fluid environment and the propellers [23], there is still a significant gap between the real motion characteristics and the simulation results based the model at high frequencies and amplitudes [24].

The EPFL CREATE lab has proposed an algorithm based on singular value decomposition (SVD) that combines simulations and experimental data to reduce the error between simulations and reality [25,26]. This approach provides good compensation for the low modeling accuracy caused by the complex coupling between flexible structures and fluids. Data-driven approaches to improve the simulation accuracy based on data show significant potential [27]. The main problem with this approach currently is the high time cost of the experimental tests and calculations required for data-driven methods. Through comparisons of the experimental and simulation results, we found that the values of the hydrodynamic coefficients had a significant influence on the simulation accuracy. Therefore, we calculated accurate values of the hydrodynamic coefficients ( Cn ) based on experiments. Furthermore, to reduce the discrepancies between the experimental and simulated results caused by the complex interactions between the fluid and solid, an novel Cn-SVD data-driven model was used to correct the simulation results and achieve more accurate predictions. This approach significantly improved the accuracy and reduced the experimental costs. With the swimming speed as the optimization target, we obtained accurate optimal control parameters by this method and verified them experimentally.

The contributions of this paper are summarized as follows: (i) A new series of FPJPs and a swimming robot platform for testing were designed. The nonlinear compliance at the joints of the paddles allowed them to generate significant net thrust via symmetric driving during the RS and PS. (ii) A dynamics model applicable to the FPJPs with different numbers of joints was developed. The simulated system was validated experimentally, and we also investigated the relationship between the number of flexible joints with nonlinear compliance and the net thrust. (iii) An novel data-driven model was developed that could be combined with the model’s simulated data to obtain accurate optimal parameters in multi-objective designs with low experimental costs. The straight swimming speeds and yaw angle distributions of the prototype robot equipped with paddles of different numbers of flexible joints and a prototype robot with the optimal control parameters before and after using the new data-driven model were analyzed based on statistics of the experimental data. This analysis verified the usefulness of the data-driven model for the multi-objective design of the oscillation amplitude and frequency of the flexible propellers.

The paper is organized as follows. In Section 2, the FPJP’s design, prototyping, and principles are described in detail. Section 3 proposes a dynamics model based on PRBM, which is applicable for different numbers of flexible joints of the FPJP, and the calculations of the hydrodynamics of the FPJP using blade element theory are presented. The simulation system built based on Simulink is analyzed and validated based on experimental results. In Section 4, an novel data-driven model for improving the simulation accuracy is proposed. In Section 5, the experimental platform for testing the thrust is presented, and a diving-beetle-like swimming robot (DBSR) is designed. Section 6 presents the experimental results, and the relationship between number of flexible joint and the propulsion ability is verified. Then, the data-driven model is verified to improve the accuracy of the simulation model by examining the DBSR swimming velocity. Finally, in Section 7, the work is summarized.

## 2. Flexible Passive Joint Paddle (FPJP) Design

This section describes the design and prototype of the proposed FPJP. The main goal of a swimming robot in generating a net thrust using the paddle stroke action is to maximize the total thrust in the PS and minimize the hydrodynamic drag in the RS [28,29,30]. For the swimming robot to achieve this goal, three FPJPs with different numbers of flexible joints were designed. The paddles used a servo motor as the driving source, and they consisted of a rigid layer made of PA66 (purple) and a flexible layer made of silicone (green). The details of materials are shown in Table 1.

**Figure 2:**
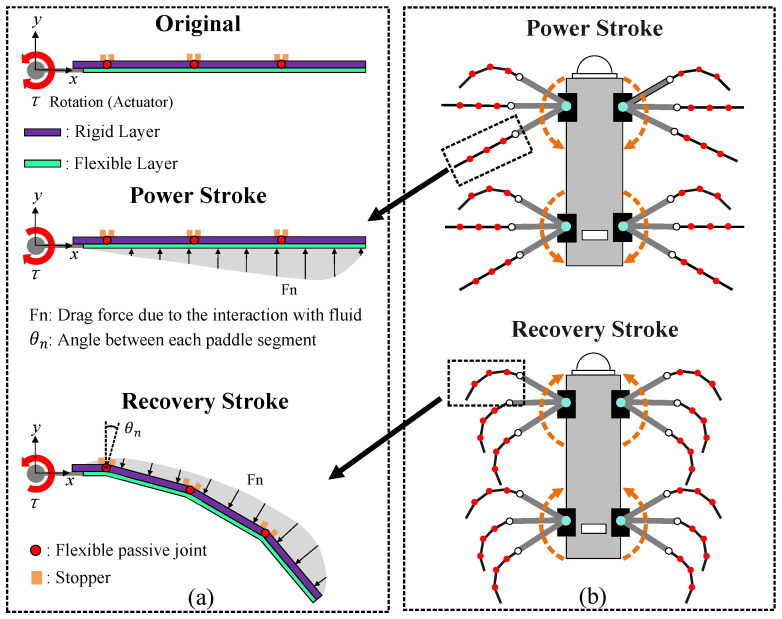
Schematic diagram of flexible passive joint paddles (FPJP) design. (**a**) Principle of the FPJP with three flexible joints (FP3JP, abbreviations for FPJPs with one and two flexible joints are defined similarly). (**b**) Morphological changes of the FPJP during the power stroke and recovery stroke on swimming robot platform.

Figure 2a shows the working principle of the FPJP with three flexible joints. The rigid layers are independent of each other, and they are connected by the flexible joints. Each flexible joint has a raised stopper on both sides. In the PS, the servo motor rotated counterclockwise, and the hydrodynamic forces drove each segment of the rigid layer together, maintaining the “flat” state through the contact of the stoppers. The generated hydrodynamic force was limited to the horizontal plane, maximizing the thrust. In the RS, the servo motor rotated clockwise, and the hydrodynamic direction drove the rigid plate segments to different angles. The resistance profile rapidly becomes smaller, which minimized resistance. Figure 2b shows the morphological changes of the FPJP during the PS and RS and the paddles with one, two, and three flexible joints (denoted as FP1JP, FP2JP, and FP3JP, respectively). The nonlinear compliance of the paddles can be passively set by the servo system based on the RS and PS during the motion cycle of the symmetric drive. The length ( $l_{2}$ ) and width (*w*) dimensions of the rigid layer and the flexible layer were fixed for all types of FPJPs at 0.105 and 0.040 m, respectively. The thickness of the flexible layer was 0.0006 m to ensure experimental durability.

## 3. Dynamics Modeling and System Identification

One of the focuses of this study was to analyze and compare the morphological changes of paddles with different numbers of flexible joints within the RS and PS and their relationship to the propulsive capacity. To this end, we develop a dynamics model applicable for an FPJP with *n* flexible joints (FPnJP, n=1,2,3⋯ ). The environment in which the FPJP operates is considered to be an inviscid, incompressible fluid. Blade element theory is used to calculate the hydrodynamic forces generated by the FPJP.

### 3.1. Dynamics Modeling

**Figure 3:**
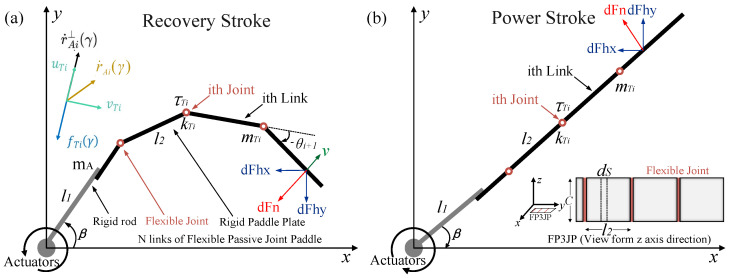
Dynamics model of the flexible passive joint paddle (FPJP). (**a**) Top view schematic of the FPJP in the recovery stroke. (**b**) Top view schematic of the FPJP in the power stroke.

The pseudo-rigid body model (PRBM) [31] theory sets up the flexible structure as a pseudo-rigid linkage connected in series with torsion springs, which maintains a high degree of consistency with the nonlinear compliance joints of the flexible propeller we designed and the multi-joint structure with high hinge connections in the legs of the diving beetle [32]. The FPJP acquires motion through the deflection of the elastic member. The FPJP rows periodically in the xy plane, as shown in Figure 3a,b. It bends and deforms through interactions with water. The compliance of the joint position varies nonlinearly, and the flexible mechanism can be accurately modeled as a rigid body mechanism in the nonlinear range [33]. Based on the PRBM, we model the FPJP as a pseudo-rigid linkage structure and set each segment of the rod to be connected in series with torsion springs, setting the mass of each segment of the paddle to be located at the end of each linkage. The fluid is assumed inviscid. The motor is controlled by the following equation to achieve the desired swing angle variation:

(1) $$\beta =A\sin\left(2\pi ft\right)+\theta_{0},$$

where β is the rotation angle of the rigid rod, $\theta_{0}$ is the offset of the rigid rod, and *A* and *f* are the amplitude and frequency of the oscillation of the rigid rod section, respectively. Figure 3a shows how the FPJP and the rigid active rod are modeled. $l_{1}$ is the length of the rigid rod section, and $l_{2}$ is the length of each joint section. *N* is the number of joints of the FPJP. $k_{T_{i}}$ is the stiffness of the *i*th joint. $\theta_{i}$ is the rotation angle of the *i*th joint. $m_{T_{i}}$ is the mass of the *i*th joint. We denote the set of joint angles by the vector *Q*:

(2) $$Q={\left[q_{1},q_{2},\ldots ,q_{N+1}\right]}^{T}={\left[\beta ,\theta_{1},\theta_{2},\ldots ,\theta_{N}\right]}^{T}.$$

$v_{T_{i}}$ and $u_{T_{i}}$ are the unit vectors parallel and perpendicular to the *i*th joint, respectively. In the xy coordinates, the position and velocity of the end of the rigid active rod are denoted as

(3) $$r_{R}\left(l_{1}\right)=\left[\begin{array}{c} l_{1}\cdot \cos\beta \\ l_{1}\cdot \sin\beta \end{array}\right],$$

(4) $$\begin{array}{c} {\dot{r}}_{R}\left(l_{1}\right)=\left[\begin{array}{c} -l_{1}\cdot \sin\beta \\ l_{1}\cdot \cos\beta \end{array}\right]\;\;\dot{\beta }=\left[\begin{array}{c} J_{A_{x}}^{P}\\ J_{A_{y}}^{P} \end{array}\right]\dot{\beta }. \end{array}$$

The position of any point on the *i*th linkage of the FPJP, $r_{Ai}\left(\gamma \right)$ , can be expressed as

(5) $$r_{Ai}\left(\gamma \right)=\left[\begin{array}{c} l_{1}\cdot \cos\beta +l_{2}\sum_{j=0}^{i-1}\cos\left(\sum_{z=1}^{j+1}q_{z}\right)\\ +\gamma \cos\left(\sum_{z=1}^{i+1}q_{z}\right)-l_{2}\cos q_{1}\\ l_{1}\cdot \sin\beta +l_{2}\sum_{j=0}^{i-1}\sin\left(\sum_{z=1}^{j+1}q_{z}\right)\\ +\gamma \sin\left(\sum_{z=1}^{i+1}q_{z}\right)-l_{2}\sin q_{1} \end{array}\right].$$

γ is the distance between a specific point and the *i*th joint, where i=1,2,⋯,N . The first and second derivatives of the position Equation (5) give the velocity ${\dot{r}}_{Ai}\left(\gamma \right)$ and the acceleration ${\ddot{r}}_{Ai}\left(\gamma \right)$ of any point on the *i*th joint of the FPJP, respectively. The perpendicular projection of the velocity on the *i*th joint is as follows:

(6) $${\dot{r}}_{Ai}^{⊥}\left(\gamma \right)=\left({\dot{r}}_{Ai}\left(\gamma \right)\cdot u_{Ti}\right)u_{Ti}.$$

According to the blade element theory, the differentiation of hydrodynamic force on each point of the paddle can be described as

(7) $$dF_{n}\left(S,t\right)=-0.5Cn\rho {\left({\dot{r}}_{i}^{⊥}\right)}^{2}wdS,$$

where the paddle’s width is denoted as *w*, Cn is the hydrodynamic coefficient, which is usually set as a constant in the simulation model [34], and ρ is the liquid density. We calculated it by combining blade element theory with data from mechanical sensors to obtain more accurate hydrodynamic coefficients in different cases, as discussed below.

By considering both the rigid active rod and the FPJP (with *N* number of joints), the mechanism has N+1 degrees of freedom. We use the Lagrangian method to model the dynamics of the FPJP. The Jacobian matrix, which contains only one non-zero column, corresponding to contributions of the rigid rod, can be obtained as follows:

(8) $$J_{A}^{P}=\overset{N+1}{\overset{︷}{\left[\begin{array}{cccc} J_{Ax}^{P} & 0 & \cdots & 0\\ J_{Ay}^{P} & 0 & \cdots & 0 \end{array}\right]}}.$$

According to ${\ddot{r}}_{Ai}\left(\gamma \right)$ , the Jacobian matrix $J_{Ti}^{P}$ , where the column contributions have been taken into account up to the ith link of the FPJP, can be obtained as Equation (9), where *i* = 1, 2, ⋯, *N*.

(9) $$J_{Ti}^{P}=\overset{N+1}{\overset{︷}{\left[\begin{array}{cccccc} J_{Ax}^{P}-l_{2}\sum_{z=2}^{i+1}\left(\sin\left(\sum_{j=1}^{z}q_{j}\right)\right) & -\sum_{z=2}^{i+1}\left(l_{2}\sin\left(\sum_{j=1}^{z}q_{j}\right)\right) & -\sum_{z=i+1}^{i+1}\left(l_{2}\sin\left(\sum_{j=1}^{z}q_{j}\right)\right) & 0 & \cdots & 0\\ J_{Ay}^{P}+l_{2}\sum_{z=2}^{i+1}\left(\cos\left(\sum_{j=1}^{z}q_{j}\right)\right) & \sum_{z=2}^{i+1}\left(l_{2}\cos\left(\sum_{j=1}^{z}q_{j}\right)\right) & \sum_{z=i+1}^{i+1}\left(l_{2}\cos\left(\sum_{j=1}^{z}q_{j}\right)\right) & 0 & \cdots & 0 \end{array}\right]}}.$$

The contribution of the column has been considered as the Jacobian matrix of the i-th joint of the FPJP can be obtained, where i=1,2,⋯,N . The mass matrix and Coriolis/centrifugal term are defined respectively as follows:

(10) $$\;\;\;\;M(Q)=\sum_{i=1}^{N}\left(m_{Ti}{\left(J_{Ti}^{P}\right)}^{T}J_{Ti}^{P}\right)+m_{A}{\left(J_{A}^{P}\right)}^{T}J_{A}^{P},$$

(11) $$\;\;\;\;V\left(Q,\dot{Q}\right)=\;\;\left[\begin{array}{ccc} \;\Gamma_{11}\;\;\;\;\;\;\;\; & \cdots & \;\;\;\;\;\;\Gamma_{1\left(N+1\right)\;\;}\\ \vdots & \ddots & \vdots \\ \;\;\;\;\Gamma_{\left(N+1\right)1}\;\; & \cdots & \;\;\Gamma_{\left(N+1\right)\left(N+1\right)\;\;} \end{array}\right]\;\left[\begin{array}{c} \;{\dot{q}}_{1}\\ \vdots \\ \;{\dot{q}}_{N+1\;\;\;} \end{array}\right],$$

where $\Gamma_{ij}$ denotes the Christoffel symbols, i=1,2,⋯,N , and j=i,⋯,N . The position vector of the point at the *j*th joint of the FPJP to the *i*th joint is

(12) rTj(γ)i=rTj(γ)−rT(i−1)(l2).

Therefore, the hydrodynamic force of the *j*th segment of the FPJP plate exerts a moment on the *i*th joint, as follows:

(13) mTj(γ)i=rTj(γ)i×fTj(γ).

The non-conservative torque for the *i*th joint of the FPJP is

(14) $$\tau_{Ti}=-k_{Ti}\theta_{i}+\sum_{j=i}^{N}\int_{0}^{l_{2}}m_{Tj}\left(\gamma \right)d\gamma .$$

The mass matrix M(Q) and the Coriolis/centrifugal term $V(Q,\dot{Q})$ are considered to be non-conservative [35], since they ignore the torque generated by the torsion spring at *i*th joint. Finally, the motion of the active rigid rod and the FPJP is expressed as

(15) $$M\left(Q\right)\ddot{Q}+V\left(Q,\dot{Q}\right)=\tau .$$

The FPJP of *n* links is modeled as *n* serially connected pseudo-rigid body links. The stiffness of each joint can be calculated by (16) as follows:

(16) $$k_{Ti}=\frac{I_{i}\pi \iota^{2}E}{L_{p}},i=1,2,\cdots ,n,$$

where $L_{p}$ is the length of the FPJP, ι is the length ratio between the *i*th joint and the paddle, *E* is Young’s modulus, and $I_{i}$ is the moment of inertia. The flapping of the paddle contains two parts, the RS and the PS [18]. The above-mentioned joint’s stiffness is a parameter in the RS, and the joint stiffness is nonlinearly increased by the stopper to maintain the “straight plate” in the PS. Table 2 shows the detailed parameters of the paddle during one propulsion cycle (three-joint paddle (FP3JP) as an example).

### 3.2. System Identification

**Figure 4:**
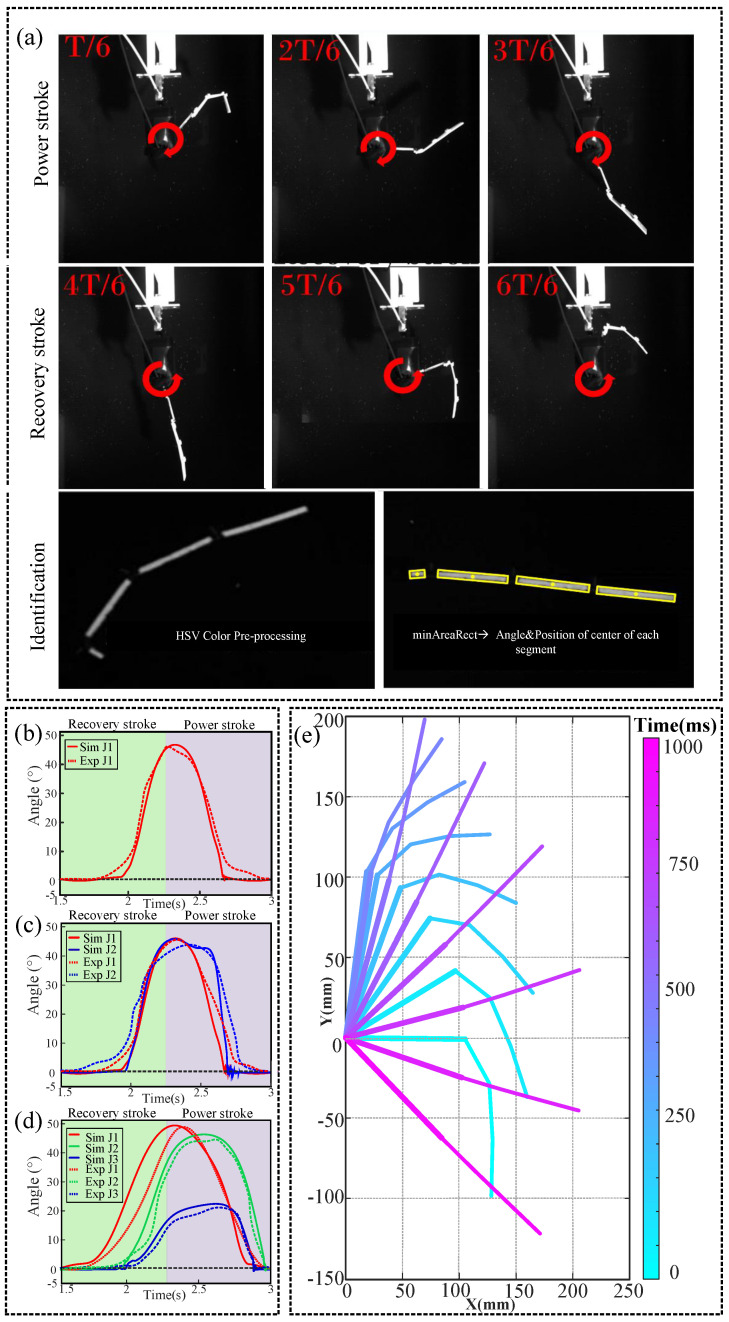
Validation of the simulation model by high-speed camera. (**a**) The motion state of the FPJP was recorded and detected using a high-speed camera. The red arrow illustrates the rotation direction of the motion. FPJP motion characteristics with a paddle oscillation frequency of 0.6 Hz and amplitude of 75°: Variations of joint angle over one movement cycle for (**b**) FP1JP, (**c**) FP2JP, and (**d**) FP3JP; solid lines represent simulated data and dashed lines represent real data. (**e**) Changes in FP3JP morphology based on simulated data during a cycle.

Simulations were constructed using Simulink based on this dynamics model. We determined the correctness of the model in our previous study by comparing the eigenvalues for the joint rotation angle, net thrust, and thrust [14]. Figure 4a shows the variation of the paddle morphology recorded using a high-speed camera for the control parameters *f* = 0.6 Hz and *A* = 75° in symmetric driving mode. The rigid layers were highlighted by modifying the image HSV (hue–saturation–value) parameters to enhance the contrast. Then, the real-time angles of the rigid layers of each segment and their positions were obtained using OpenCV’s minAreaRect function. Figure 4b–d show the simulated data (solid line) and the real data (dashed line) of the joint rotation angles obtained experimentally for the FP1JP, FP2JP, and FP3JP using the control parameters *f* = 0.6 Hz and *A* = 75° for a single cycle, with the real data being the average of the angles from five cycles. It can be seen that for different types of propellers, the simulated data and the real data had the same trends and similar values, verifying the accuracy of the dynamics model. The passive bending of the paddle within the RS reduced the area of the force profile. The fully extended and “flat” state in the PS maximized the thrust. This was consistent with the morphology captured by our high-speed camera in Figure 4a, and the accuracy of the bending angles of the joints in the simulated data was extensively verified in Figure 4b–d. With FP3JP as an example, Figure 4e shows the passive morphological change process due to the hydrodynamic forces during the motion cycle of the paddle based on the simulated data.

## 4. Optimization Method

The simplifications in the modeling and the defects in the FPJP manufacturing have led to discrepancies between the simulation and actual performance. We have experimentally verified that the simulation results accurately represent the macroscopic trends of FPJP. Section 4.1 and Section 4.2 showcase and analyze the contribution of the experimental scanning for obtaining the coefficient Cn and the SVD algorithm, respectively, in enhancing the accuracy of the underwater propulsor FPJP simulation. Section 4.3 introduces the Cn-SVD algorithm, which determines a transformation matrix using a small set of experimental data. This matrix minimizes the error between simulation and experimental data, scaling and adjusting the simulated behavior to better match the numerical and experimental results. Compared to obtaining the correct Cn directly through experimental scanning, our method significantly reduces the error between simulation and experiment and requires only a minimal amount of experimental data, thus reducing the experimental wear and processing time of the flexible propulsor. In contrast to applying the SVD algorithm as an example of a data-driven linear model, the Cn-SVD algorithm can capture the nonlinear variations in data changes more accurately with a small amount of experimental data.

### 4.1. Effect of Hydrodynamic Coefficients on Simulation

**Figure 5:**
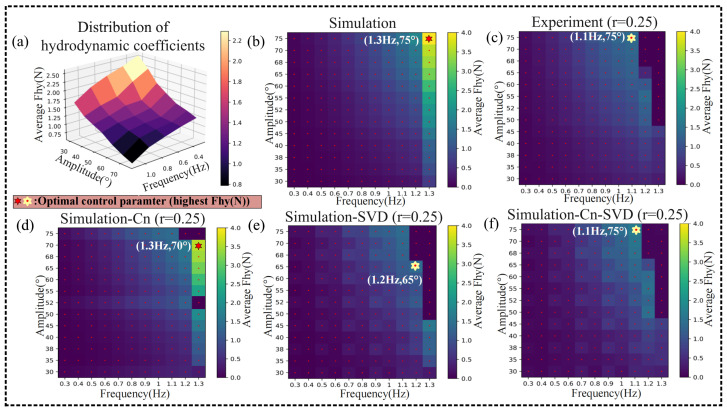
(**a**) Distribution of Cn . (**b**–**f**) Pre-transform simulation, experimental, post-transformation ( Cn , SVD, and Cn-SVD) average thrust thermograms for varying oscillation amplitude *A* and frequency *f*, demonstrating the successful error reduction between the experiment and simulated data. The symbol in each figure shows the oscillation amplitude and frequency generating the largest average force.

Many of the coefficients in the model, such as robot mass and moment of inertia, could be measured or calculated directly. Some coefficients can be easily measured by simple experiments [36,37,38,39]. For example, the drag coefficient of a robot body was measured using a force gauge [40]. However, some coefficients, such as the hydrodynamic coefficient ( Cn ) in a CFD model [41], are difficult to measure. Therefore, we identified them from experimental motion data. We collected the peak values in the time series of the tested thrust, and by studying the morphology of the FPJP, it was found that the peak value of the thrust occurred when the drag profile was at its maximum. The paddle was also fully expanded, and the drag profile size resembled the paddle area. The exact hydrodynamic coefficients for different control parameters can be obtained from the following equation:

(17) $$C_{n}=-\frac{4dF_{n}}{S^{2}\rho w{\left({\dot{r}}_{i}^{⊥}\right)}^{2}},$$

where *w* is the width of the paddle, *S* is the position of the paddle, $F_{n}$ is the drag force at the *i*th point of the paddle, and ${\dot{r}}_{i}^{⊥}$ is the perpendicular projection of the velocity with respect to the *i*th point. In the experiment, the oscillation frequency was changed every 0.1 Hz from f∈[0.3,1.3] Hz, and the amplitude was changed every 5° from $A\in [30^{\circ }$ , $75^{\circ }$ ]. Each experiment was performed five times to reduce errors. The Cn distribution is shown in Figure 5a. The data ratio *r* is defined as measuring the amount of experimental data required to transform the simulated data. The sampling of experiments corresponding to *r* depends on the range of simulated data, with uniformly equally spaced within its range:

(18) $$r=\frac{V_{e}}{V_{s}}.$$

Figure 5 exhibited the results for r=0.3 , taken uniformly over the range of control parameters. The magnitude of Cn decreased monotonically as the oscillation amplitude and frequency increased. Figure 5b–d compare the experimental data, simulated data, and simulated data with the substitution of Cn . It can be found that substituting the exact Cn captured the FPJP morphology and the unquantifiable trends in the thrust due to material limitations well. The difference between the simulated data and reality was reduced.

### 4.2. Simulation to Reality Transfer

Due to the complex coupling of flexible propeller structures and fluids, inevitably, there are differences between the simulation and experimental results due to the model assumptions and large deformations. Our model has been shown in previous studies [14] to respond accurately to real macroscopic trends, and specific differences between the simulation and experimental results must be reduced. Using limited experimental data, we aimed to determine the transformation matrix to transform the simulated results and to the experimental results. The solution developed by Arun et al. for the SVD calculation of matrices [42] showed that based on limited experimental data, we can construct a linear transformation to adjust the simulated data so that the Euclidean distance between the adjusted simulated data and the experimental data is minimized. This way, we can minimize the difference between the transformed simulations and the experiments. The simulation can be used as an experimental proxy to provide an accurate prediction [43]. We restrict our discussion to the three-dimensional case. Given a sequence simulation output values $\left\{s_{k}\right\}$ and a sequence of experimentally measured values $\left\{e_{k}\right\},k=1,2,\cdots ,N$ , we seek to find a linear transformation written in the form of

(19) $$e_{k}=Rs_{k}+T+N_{k},$$

where *R* is the rotation matrix, *T* is the translation vector, and $N_{k}$ is the noise term. We choose an appropriate loss function to be minimized:

(20) $$L=\sum_{k=1}^{N}{∥e_{k}-\left(Rs_{k}+T\right)∥}_{2}^{2}.$$

An efficient approach was developed based on the SVD method to solve this problem. To illustrate the algorithm, we first present a lemma from advanced algebra referring to Blostein and Eggert’s work [42,43].

 **Lemma 1.**  *Let $\alpha ={\left(a_{1},a_{2},\cdots ,a_{n}\right)}^{T}\in R^{n}$ , where R is a rotation of α, and T is a translation of α. Then, the centroid of α is equal to the centroid of Rα+T . i.e., if*

(21) $$R\alpha +T={\left(b_{1},b_{2},\cdots ,b_{n}\right)}^{T}.$$

*Then,*

(22) $$\frac{1}{n}\sum_{i=1}^{n}a_{i}=\frac{1}{n}\sum_{i=1}^{n}b_{i}.$$

*For better description, we give notations as follows:*

(23) $$\bar{s}≜\frac{1}{N}\sum_{k=1}^{N}s_{k};$$

(24) $$\bar{e}≜\frac{1}{N}\sum_{k=1}^{N}e_{k}.$$

(25) $$s_{k}^{C}≜s_{k}-\bar{s},\;k=1,2,\cdots ,N;$$

(26) $$e_{k}^{C}≜e_{k}-\bar{e},k=1,2,\cdots ,N.$$

By applying Lemma 1, we can remove the translation vector *T* from the loss function:

(27) $$L=\sum_{k=1}^{N}{∥e_{k}^{C}-Rs_{k}^{C}∥}_{2}^{2}.$$

The superscript *C* represents the centered version of the variables $s_{k}$ and $e_{k}$ . Specifically, $s_{k}^{C}$ is the centered simulation data and $e_{k}^{C}$ is the centered experimental data. Therefore, according to Algorithm 1, the original least squares problem is reduced to two parts: (1). Find the *R* rotation matrix such that *L* is minimized. (2). Then, obtain the translation vector as follows:

(28) $$T=\bar{e}-R\bar{s}.$$

The algorithm is given below, and here we refer to Eggert’s work [43] for more detailed illustration.
**Algorithm 1:** Least Squares Method for Simulation Revision
  Calculate the centroids $\bar{s},\bar{e}$ ; then calculate $\left\{s_{k}^{C}\right\}$ and $\left\{e_{k}^{C}\right\}$ .  Calculate $H\leftarrow \sum_{k=1}^{N}s_{k}^{C}{\left(e_{k}^{C}\right)}^{T}$ .  Calculate the SVD of *H*, i.e., $H=U\Lambda V^{T}$ .  Calculate $X=VU^{T}$ .  If det(X)=1 , then the **rotation matrix** R=X .  If det(X)=−1 , the algorithm fails and a RANSAC-like technique is required [44].  Calculate the **translation matrix** $T=\bar{e}-R\bar{s}$ .  **Return** *R*, *T*


The simulated data set *S* should be concatenated with an end column of 1’s with dimensions of [N×1] , i.e., $S=\left\{\left[s_{k},1\right]\right\}$ . We can consider *S* to be a matrix and represent these operations as an augmented transformation matrix *M*, with the form of

(29) $$M=\left[\begin{array}{cc} R & T\\ 0 & 1 \end{array}\right].$$

Finally, the transformed simulation is given by $S_{T}={\left(MS^{T}\right)}^{T}$ . The simulated data was processed by the SVD algorithm, and it can be found that the SVD algorithm significantly reduced the experimental error (Figure 5e). In this model, while the quantity of experimental data used should be minimized, the dataset must span the space of control parameters of interest, and the simulation data should cover the same range. Additionally, predictions made using computational transformations should be confined within the same domain of interpretation to avoid extrapolation errors. Since the experimental data were used to identify a transformation matrix that maps the simulated data to the experimental data with the lowest error to achieve the effect of reducing the simulated data error, the experimental data set should span the control parameter space of interest, and the simulated data should span the same range. Among them, the experimental data involving a nonlinear change trend can significantly reflect the advantages of our proposed algorithm compared with the previous linear model (SVD algorithm). Using 25% (r = 0.25) of the simulated data for the transformation of the experimental data allowed the total error of the transformed simulated data to be reduced to 6.73%.

### 4.3. Novel Semi-Empirical Data-Driven Model

**Figure 6:**
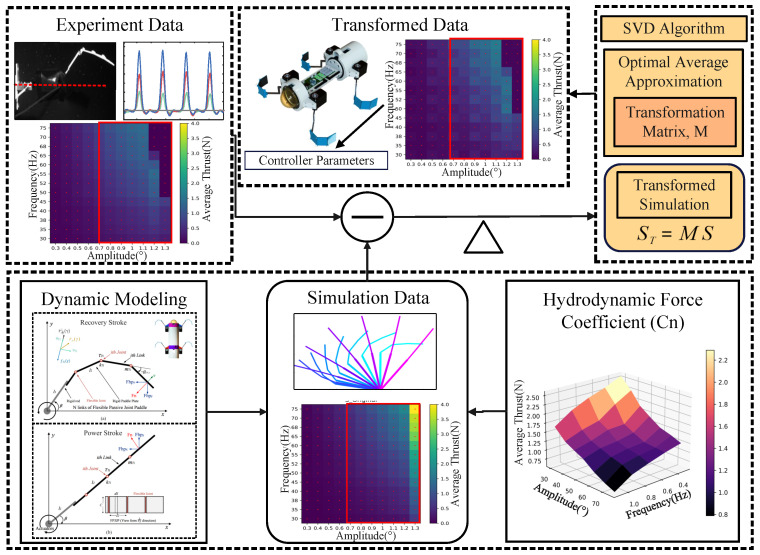
Overall process and logic of the work.

**Figure 7:**
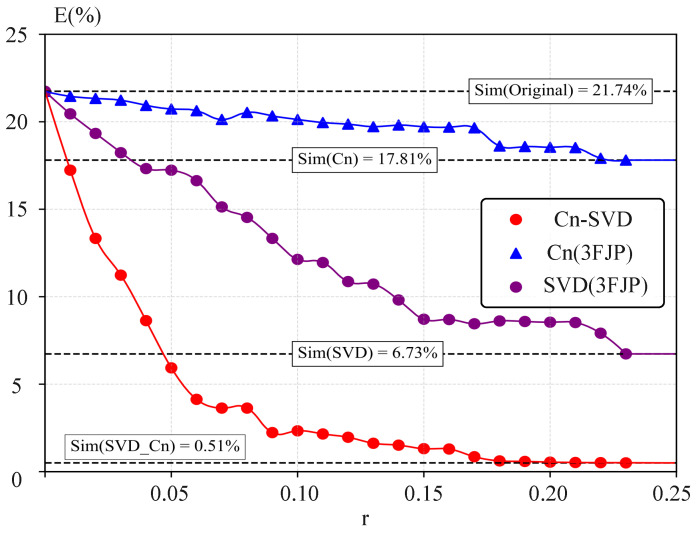
Comparison of the average error with the experimental data for real ratios of r∈[0,0.25] : simulated data, simulated data with Cn , simulated data after SVD transformation, and simulated data of the novel Cn-SVD combined data-driven model.

In Section 4.1 and Section 4.2, the contributions of the hydrodynamic coefficient method and the SVD algorithm to the accuracy improvement of the FPJP simulations are presented and analyzed. Both approaches allow efficient compensation of the control parameters by combining real data in cases where the macroscopic trends of the simulated and real data are similar. Compared to the traditional compensation from the perspective of tuning modeling [45,46,47], the direct optimization of the control parameters with experimental data has better adaptability. It effectively quantifies the complex coupling of the flexible structure to the fluid environment. For underwater vehicles, improving the accuracy of simulation data can contribute to the multi-objective optimization of propulsion (e.g., swimming speed of underwater vehicles under different structural and control parameters, mechanical efficiency of propulsion, etc.) [48,49,50] , which has a broad research potential. However, the optimization algorithms and data acquisition usually used by researchers require considerable computational hardware and have high time costs. We developed a data-driven model with low experimental data requirements by combining the hydrodynamic coefficient ( Cn ) method and SVD algorithm. A large amount of multivariate simulation data was accurately transformed by effectively utilizing a small number of experiments. The overall process and logic of the work is shown in Figure 6. In calculating simulation data, compared to directly inserting the real fluid dynamic coefficients ( Cn ) obtained from experiments, the Cn-SVD algorithm requires only 5% of the experimental data to achieve a lower overall error, as demonstrated in Figure 7. Figure 5d,f and Figure 7 present a performance comparison between the Cn-SVD algorithm and the direct interpolation of real hydrodynamic coefficients. Compared to the data-driven linear model constructed using the SVD algorithm, the Cn-SVD algorithm more accurately captures nonlinear changes in the data with a smaller amount of experimental data, as evidenced in Figure 5e,f and Figure 7.

Since the experimental data are used to identify the transformation matrix that maps the simulated data to the experimental data with the lowest error thereby achieving the effect of reducing the simulated data error, the experimental dataset should span the control parameter space of interest and the simulated data should span the same range. In particular, experimental data involving nonlinear trends can significantly demonstrate the advantages of our proposed algorithm over previous linear models (SVD algorithm). For example, when the frequency range of the simulated data is increased above 1.1 Hz, a sudden drop in thrust (a nonlinear trend) is observed in the corresponding experiments, which cannot be captured by the simulation model itself. In this case, it is necessary to correspond the range of the simulated data to the range of the experimental data to compare the effect of the SVD algorithm with that of the Cn-SVD algorithm in a more intuitive way.

The data ratio *r* is defined as measuring the amount of experimental data required to transform the simulated data, as shown in Equation (17). $V_{e}$ is the amount of experimental data, and $V_{s}$ is the amount of simulated data. The differences between the simulated and experimental data are captured using the average total error *E*:

(30) $$E=\sum_{i=1}^{n}\sum_{j=1}^{m}\frac{e_{ij}}{X_{ij}},$$

where $e_{ij}$ is the difference between simulated and experimental data at each frequency and amplitude, and $X_{ij}$ is the experimental data at each frequency and amplitude. Each increase in *r* allows the experimental data to be uniformly distributed within the simulated data. The effect of Cn combined with the SVD algorithm in the range of r∈[0,0.25] on the average total error *E* between the simulated and experimental data was counted. The results showed that the new algorithm could achieve high-accuracy approximations from simulations using limited experimental data. The improvement of the simulation accuracy by replacing Cn is limited. However, the use of the SVD algorithm significantly improved the accuracy, reducing the amount of experimental data required considerably (Figure 5f). The value of *E* for the simulated and experimental data was 21.74%. Using the r∈[0,0.25] range of Cn reduced the *E* to 17.81%. The error reduction was not significant, but as shown in Figure 5b–d, using the correct Cn in the critical range can correctly capture the target amount of the trend with a strong orientation. Using the SVD algorithm allowed *E* to be reduced to 6.73% in the r∈[0,0.25] range. However, as shown in Figure 5b,c,e, using only the SVD algorithm did not provide a good approximation of the large variation trend in the critical range of the target quantity, which was detrimental to the search for the optimal control parameters (or other multivariate function optimization problems). The Cn-SVD algorithm proposed in this paper uses a small amount of experimental data to determine a transformation matrix that maps simulated data to experimental data with minimal error. The optimization effect is reflected in the fact that through this transformation, we can not only optimize the simulation data to more accurately reflect the experimental observations, but also gain insight into the specific effects of design and control parameters on performance, for example, this approach allows us to design robots optimized for control parameters targeting the highest thrust. We validate the experimental results of control parameter optimization targeting the highest thrust by measuring the swimming speed in Section 6.2.

The novel Cn-SVD data-driven model could accurately capture the variation trend of the target value and approximate the realistic data using fewer experiments. *E* could be reduced to 0.51% within the r∈[0,0.25] range, and the trend of the simulated data was also accurate. The detailed data are shown in Figure 7. When an experimental data volume of 25% of the simulated data volume was used, *E* was reduced to 0.51%. For a more complete evaluation of the effects of each method, we analyze in detail the maximum error as well as the average error of the simulated and experimental data for the case r∈[0,0.05,0.10,0.15,0.20,0.25] in Table 3. Statistical results show that our proposed Cn-SVD algorithm is equally effective in reducing the maximum error over the range of simulated data.

## 5. Experimental Setup and Swimming Robot Prototype

In this study, two experiments were conducted. Experiment A verified the conclusion based on the dynamics model that the increase in the number of flexible joints on a propeller moving in rowing mode was beneficial for enhancing the net thrust with an underwater force test bed. In Experiment B, a DBSR was designed with two pairs of FPJP modules that could carry different types of FPJPs and take different control parameters *A* and *f* for a symmetric driving mode. The DBSR was equipped with an inertial measurement unit (IMU) for the feedback yaw angle and a communication module for remote control.

### 5.1. Experimental Setup

**Figure 8:**
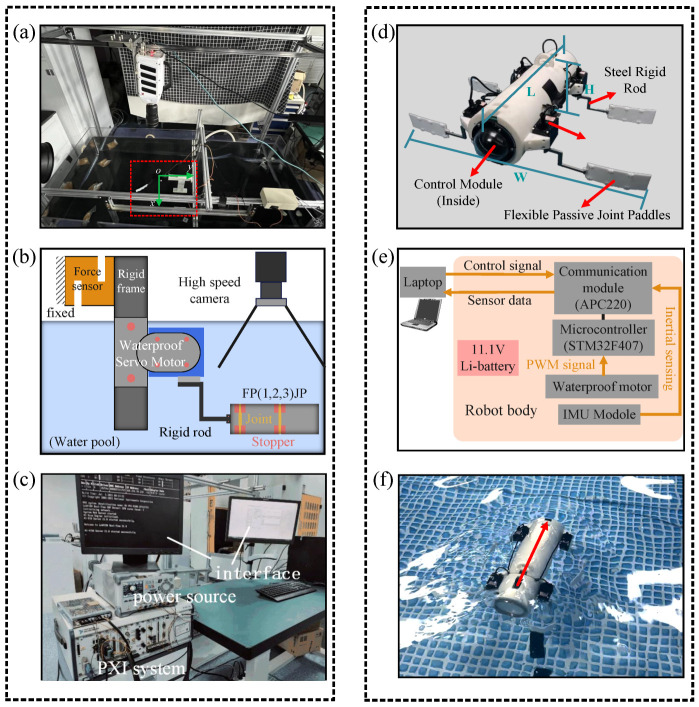
Experimental setup. (**a**) High-speed camera with a force measurement system. (**b**) Schematic diagram of the composition of the force measurement system. (**c**) User interface and PXI system. (**d**) Design specifications of the diving-beetle-like swimming robot (DBSR). (**e**) Hardware configuration inside the DBSR body. (**f**) Swimming behavior of the DBSR observed from the top view.

An experimental test bed for collecting experimental data on the thrust force generated by the FPJP in the *y*-direction ( $F_{hy}$ ) was developed, as shown in Figure 8a. The FPJP was activated in a water tank using the XW540-T40-R waterproof servo. To minimize edge effects, we tested each experiment at the center of a 2.0 m (length) × 1.3 m (width) × 0.7 m (height) acrylic tank, with the FPJP (0.105 m (length)) positioned far enough away from the tank wall. The servo motor driving the FPJP was connected to a load cell (Model: DYLY-102) via a connector, and a force gauge was used to measure the $F_{hy}$ (N) generated by the FPJP (Figure 8b). The load cell was calibrated to ensure that no torque was applied to the force-measuring element, and then it was connected to an amplifier whose output was connected to the data acquisition board on the PXI system, with a sampling rate of 1000 Hz. A programmable power supply (Model: DP832A) ran the computer with a user interface programmed in LabVIEW (Figure 8c).

### 5.2. Design of Diving Beetle Swimming Robot

The DBSR had four independent FPJP modules, as shown Figure 8d. Each paddle was connected to a stainless-steel rigid rod to enhance the range of motion. The swimming robot was powered by a waterproof servo motor (XW540-T140-R), which supported the real-time speed adjustment and torque feedback. The design specifications are detailed in Table 4. This swimming robot measured 0.426 m in length, 0.529 m in width, and 0.110 m in height, with a total weight of 2.5 kg. To ensure that the robot operated underwater without being affected by the water, the sealed compartment was inside the rigid head and made of an organic glass material. The diameter of the sealed compartment was 0.11 m, the length was 0.20 m, and the thickness was 0.005 m. The sealed compartment passed the sealing test to ensure the robot could operate safely underwater for a long period. The control module consisted of a microcontroller (STM32F407), an IMU (MPU6050, used to obtain the direction and angular velocity), a 7000-mAh lithium battery, and a wireless communication module (APC220). The relationship between these modules is shown in Figure 8e. The swimming robot was designed to have neutral buoyancy, with an overall density close to that of water (Figure 8f). During the experiment, the IMU installed in the DBSR was used to obtain direct information, and the data was transmitted back to the computer in real-time through a wireless communication module. The oscillations driven by the servo motor followed a sinusoidal wave pattern. All the experiments were started when the water surface was close to still to reduce experimental errors.

**Table 4:** Design specifications of the robotic diving beetle.

Component	Parameter	Value	Unit
Body	Mass	2.5	kg
	Length	0.426	m
	Width	0.569	m
	Height	0.110	m
FPJP	Servo arm length ( $l_{p}$ )	0.010	m
	Mass ( $m_{p}$ )	0.029	kg
	Length ( $L_{p}$ )	0.105	m
	Width	0.040	m
Hardware	Microcontroller	STM32F407	
	IMU	MPU6050(JY61)	
	Servomotors	XW540T40R	
	Communication module	APC220	
	Battery	11.1	V

## 6. Results and Discussion

### 6.1. Experiment A

The effect of the number of flexible joints on paddle propulsion was experimentally investigated. The total length (0.105 m) and the width (0.040 m) of each type of FPJP were the same. We tested the propulsion performances of three paddles with one, two, and three equal segments in the range of oscillation amplitudes ( $A\in [30^{\circ },75^{\circ }]$ in 5° increments) and frequencies ( f∈[0.3,1.1] Hz in 0.1-Hz increments). Three steadily varying thrust time series cycles were recorded with a recording frequency of 1000 Hz. To better visualize the asymmetric thrust, we used the cycle-averaged thrust $F_{a}$ to measure the propulsive performance of the paddle:

(31) $$F_{a}=\frac{\int_{0}^{t}F\left(t\right)dt}{t}.$$

**Figure 9:**
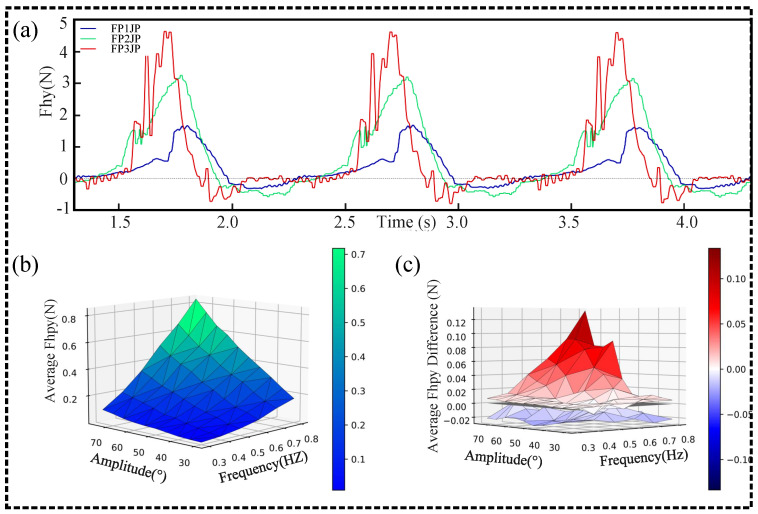
(**a**) Representative time series from FP1JP, FP2JP, and FP3JP thrust tests. (**b**) Average thrust of the two-joint paddle with multiple control parameters (amplitude and frequency). (**c**) Distributions of the differences between the average thrust values of the two-joint paddle and those of the three-joint (red) and one-joint (blue) paddles.

A representative thrust time series obtained from the underwater force sensor is shown in Figure 9a. From these experimental thrust time series (and the corresponding simulated thrust time series), we obtained the instantaneous thrust time series and the average thrusts for the three types of FPJPs being driven for about 8 s. We can observe a positive correlation between the thrust and the number of joints. Subsequently, the average thrust( $F_{hy}$ ) distribution of the FP2JP was calculated for different amplitudes ( $A\in [30^{\circ },75^{\circ }]$ in 5° increments) and frequencies ( f∈[0.3,1.3] Hz in 0.1-Hz increments). Figure 9b,c show the differences in the average thrust ( $F_{hy}$ ) between the FP1JP (blue envelope), FP3JP (red envelope), and FP2JP paddles for various values of the control parameters. It can be observed that the FP3JP exhibited a better performance than the FP2JP, while the FP1JP yielded less net thrust than the FP2JP in all cases. The rapid deformation of the FPJP joints led to a change in the drag curve, resulting in more net thrust. Using a symmetric control mode, a standard plate of equal size produced an approximately symmetric thrust with a net thrust close to zero. In contrast, with a flexible paddle with an asymmetric structure, such as FP3JP, the positive average thrust was almost five times higher than the negative average thrust. Therefore, we concluded that increasing the number of flexible joints allowed the paddles to generate an effective thrust using simple symmetric actuation and that the number of flexible joints was positively correlated with the propulsive capacity.

In previous simulations and experiments, with a certain mass of paddles, we have tried FPJP with 4 and 5 joints, and the experimental measurements found that the static thrust increase becomes slow when there are too many joints. Meanwhile, the difficulty of optimizing the physical production of FPJP will increase. Considering that there is still a large workload in optimizing control parameters, we only extended the number of joints to 3 joints, that are sufficient to verify the relationship between the number of joints and static thrust in this work.

### 6.2. Experiment B

The improvement of the accuracy of the Cn-SVD algorithm on the simulated data in Section 4.3 was verified through swimming experiments using the optimal control parameters in the range of the simulated data before and after the transformation, respectively, for swimming speed measurements. In addition, demonstrate how these modular asymmetric paddles can be combined to create an effective soft swimming robot. The robot swam in a tank with dimensions of 5.49 (length) × 2.74 (width) × 1.32 (height) m. The average swimming speed of the robot was measured by a stopwatch and soft ruler mounted on the side of the tank, while we had a researcher standing quietly in the pool using a camera to assist in recording the robot’s swimming state. We used a swimming robot equipped with four FPJPs symmetrically. Each straight swimming experiment lasted for 10 s. We used the IMU module to acquire the yaw angle of the swimming robot with a collection frequency of 0.1 s, and we measured the effective straight swimming distance of each swimming robot to obtain the average speed.

**Figure 10:**
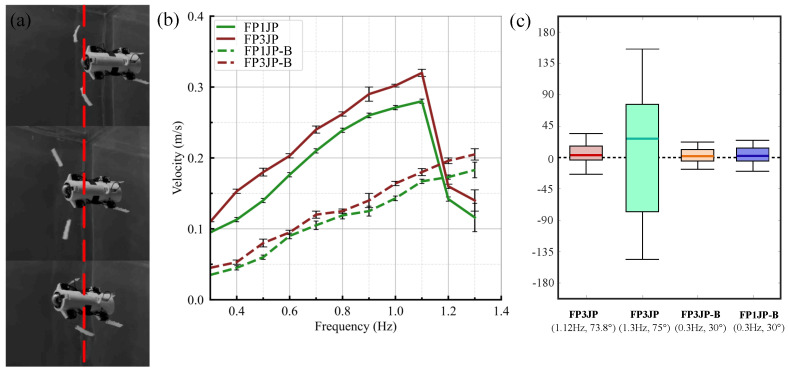
(**a**) Experiment of DBSR while swimming. The red dashed line is the reference line. (**b**) Comparison of robot velocities with FP1JP and FP3JP for different frequencies and amplitudes of 75° (solid lines) and 30° (dashed lines). (**c**) Yaw angle distribution of DBSR swimming straight with different control parameters.

First, the average speed and yaw angle distributions of the DBSR equipped with FP1JP and FP3JP, respectively, in the swing frequency range of f∈[0.3,1.3] Hz for the case with a swing amplitude of *A* = 75° were tested, and the straight swimming experiments were performed five times for each control parameter. This range of motion was chosen for the test taking into account the range of extreme forces that can be withstood using the materials used to make the paddles. The swim speed distribution of the DBSR is shown as a solid line in Figure 10b. The experimental data showed that the FP3JP had the fastest swim speed of 0.323 m/s in the case with *A* = 75° and *f* = 1.1 Hz (the control parameters were set approximately equal to the best control parameters of the simulated data obtained using the Cn-SVD algorithm). The average speed increased monotonically until *f* = 1.1 Hz, and then it decreased monotonically after that peak. The same trend of the velocity variation was observed when the DBSR was equipped with FP1JP. The control parameters *A* = 75° and *f* = 1.3 Hz in the simulation model had a larger average $F_{hy}$ , but the actual speed was only 0.142 m/s. This was because the stability of the propeller morphology change decreased after the control parameters exceeded the critical values. The thrusts generated on both sides of the DBSR were asymmetric, thus leading to a significant increase in the yaw angle and a decrease in the effective straight travel distance (Figure 10c). The yaw angle distribution range and the average value at *A* = 75° and *f* = 1.3 Hz are significantly higher than those at (*A* = 75° and *f* = 1.1 Hz) and other baseline data. These could not be simulated by the dynamics model effectively. After transforming the simulated data using the Cn-SVD algorithm, straight-line swimming tests were performed using the optimal controller parameter values (corresponding to the maximum average thrust in the simulated data). The results show that the DBSR swims using the optimal controller parameters of the transformed simulated data (*A* = 75° and *f* = 1.1 Hz) with an 89.99% reduction in yaw angle and a 2.275-fold increase in speed compared to using the optimal controller parameters before the transformation (*A* = 75° and *f* = 1.3 Hz). This validates that the novel data-driven model proposed in this paper helped researchers understand the specific effects of design and control parameters on the performance of underwater flexible propellers.

Then, the average speed and yaw angle distribution of the DBSR were tested for the case of an oscillation amplitude of *A* = 30° (Baseline) and the oscillation frequency of f∈[0.3,1.3] Hz with the FP1JP and FP3JP. Straight swimming motion experiments were conducted five times for each control parameter. From the dashed line in Figure 10b, it can be observed that the mean velocity continued increasing monotonically, and the swimming speed of DBSR with the FP3JP was always higher than that with the FP1JP, which verified the correctness of the conclusion of Experiment A. The optimal control parameters (highest propulsion force in simulation, *A* = 75° and *f* = 1.3 Hz) before transform resulted in asynchronous changes in the propeller paddle morphology and an asymmetric propulsion force ( $F_{hy}$ ) distribution, significantly increasing the yaw angle variation. The detailed directional swim speed and yaw angle statistics are shown in Table 5. It can be found that the optimal control parameters obtained from the simulated data transformed by the Cn-SVD model did not produce excessive yaw angles while moving at high speeds (the propulsor maintained a stable period of morphological change), proving our proposed algorithms practicality.

## 7. Conclusions

This study proposed and validated the performance of FPJP utilizing passive flexible joints to provide greater net thrust and higher propulsive efficiency, and developed a swimming robotic platform for testing. Then, the dynamics of the FPJP were modeled using the pseudo-rigid body model and blade element theory. The simulation model accuracy was verified. Next, a novel data-driven model (Cn-SVD model) with the combination of experimental hydrodynamic coefficients and the SVD algorithm was proposed to improve the accuracy of large-scale simulated data when utilizing limited experimental data. This approach could reduce the total error between the simulated and experiment data to 0.51%. Finally, an underwater thrust measurement system was built for Experiment A. The results showed that the propulsive capability was positively correlated with the number of flexible joints of the FPJP. The straight swimming speed of the swimming robot in different cases was tested in Experiment B. The transformed simulation data using the Cn-SVD algorithm provided an accurate relationship between the average propulsion force and the control parameter. Experiment B showed that the swimming robot using the optimal control parameter of the transformed simulation data (1.1 Hz, 75°) was the fastest, with an effective swimming speed of 0.323 m/s, was 2.275 times faster than the fastest swimming speed using the optimal control parameter of the pre-transfer (0.3 Hz, 30°), and was 3.3 times faster than the baseline (0.3 Hz, 30°). This proved that the Cn-SVD model could effectively approximate reality based on limited experimental data. Accurate simulation data helped us to understand the specific effects of design and control parameters on the performance of underwater flexible thrusters.

## 8. Future Plans

In the next work, details will be given on how topology optimization methods can develop fully flexible structures and improve stress distribution during robot motion [51,52]. In the current FPJP design, despite the high flexibility, localized stress concentrations do exist at the flexure joints during the bending motion. Our future work will explore how these stress concentrations can be mitigated through design improvements, with possible approaches including improved joint design, use of new materials, and adoption of topology optimization techniques.

## Figures and Tables

**Table 1:** Technical details of the used materials.

Material	Parameter	Value	Unit
PA66	Density	1.14	g/cm^3^
	Melting point	260	°C
	Young’s modulus	2.7	GPa
Silicone	Density	1.2	g/cm^3^
	Melting point	250	°C
	Young’s modulus	2	MPa

**Table 2:** Parameters of the FP3JP.

Parameter	Meaning	Value	Unit
$l_{1}$	Rigid rod length	105	mm
$l_{2}$	Paddle segment length	35	mm
*w*	Paddle width	40	mm
Cn	Hydrodynamic coefficient	1	
$m_{Ti}$	Mass of each paddle segment	10	g
$m_{A}$	Mass of rigid rod	16	g
$k_{T1}$	Stiffness of the first joint	0.032	N·m/rad
$k_{T2}$	Stiffness of the second joint	0.031	N·m/rad
$k_{T3}$	Stiffness of the third joint	0.031	N·m/rad

**Table 3:** Comparison of Maximum, Average, and Maximum Errors for Different Methods.

	Cn		SVD		Cn-SVD	
	**Maximum Error**	**Average Error**	**Maximum Error**	**Average Error**	**Maximum Error**	**Average Error**
r = 0.00	99.86%	21.74%	99.86%	21.74%	99.86%	21.74%
r = 0.05	99.86%	20.65%	87.63%	14.87%	54.41%	6.12%
r = 0.10	98.91%	20.38%	74.42%	12.21%	34.98%	2.37%
r = 0.15	98.91%	19.47%	59.79%	8.32%	16.83%	1.59%
r = 0.20	95.43%	18.52%	56.87%	8.25%	12.64%	0.56%
r = 0.25	95.43%	17.81%	47.45%	6.73%	8.29%	0.51%

**Table 5:** Directional swimming experimental data of swimming robots.

Paddle Type	Control Parameters	Average Speed	Average Yaw Angle	Maximum Yaw Angle
FP3JP	1.1 Hz, 75°	0.323 m/s	2.73°	35.29°
FP3JP	1.3 Hz, 75°	0.142 m/s	27.29°	151.63°
FP1JP	1.1 Hz, 75°	0.279 m/s	2.73°	35.29°
FP1JP	1.3 Hz, 75°	0.126 m/s	27.29°	151.63°
FP3JP	0.3 Hz, 30°	0.093 m/s	1.57°	22.19°
FP1JP	0.3 Hz, 30°	0.082 m/s	1.47°	24.87°

## Data Availability

The datasets generated during and/or analyzed during the current study are available from the corresponding author on reasonable request.
